# Usefulness of the Fibrosis-3 index in patients with hepatitis C

**DOI:** 10.1097/MS9.0000000000003851

**Published:** 2025-09-12

**Authors:** Kazuya Kariyama, Hidenori Toyoda, Takashi Kumada, Satoshi Yasuda, Shohei Shiota, Akiko Wakuta, Kazuhiro Nouso

**Affiliations:** a Department of Gastroenterology and Liver Disease Center, Okayama City Hospital, Okayama, Japan; b Department of Gastroenterology and Hepatology, Ogaki Municipal Hospital, Ogaki, Gifu, Japan; c Department of Nursing Faculty of Nursing, Gifu Kyoritsu University, Ogaki, Gifu, Japan

**Keywords:** age, FIB-3 index, FIB-4 index, liver fibrosis, viral hepatitis

## Abstract

**Aim:**

Noninvasive liver fibrosis prediction scores are crucial for evaluating liver disease progression. It was previously reported that the newly proposed Fibrosis-3 index (FIB-3 index) is useful in cases of metabolic dysfunction-associated steatotic liver disease. In this study, we investigated whether it is useful in cases of hepatitis C, a viral liver disease.

**Methods:**

We evaluated the liver fibrosis prediction ability of both the FIB-3 and FIB-4 indices by age-group in 517 patients with treatment-naïve hepatitis C who underwent liver biopsy.

**Results:**

The FIB-3 and FIB-4 indices linearly increased with fibrosis progression in patients with hepatitis C virus infection. Receiver operating characteristic analysis revealed no significant difference between the two indices in predicting advanced fibrosis (≥F3) and moderate fibrosis (≥F2). The FIB-3 index showed significantly higher accuracy (47.8%) in predicting advanced fibrosis in patients aged ≥70 years than the FIB-4 index (28.3%) using a single cutoff value derived from the Youden index for the entire cohort.

**Conclusions:**

The FIB-3 index proved useful for predicting liver fibrosis and prognosis in patients with hepatitis C, with performance comparable to that of the FIB-4 index. Moreover, its ability to use a single cutoff value across age-groups may offer a clinical advantage.

## Graphical abstract

**Figure:**
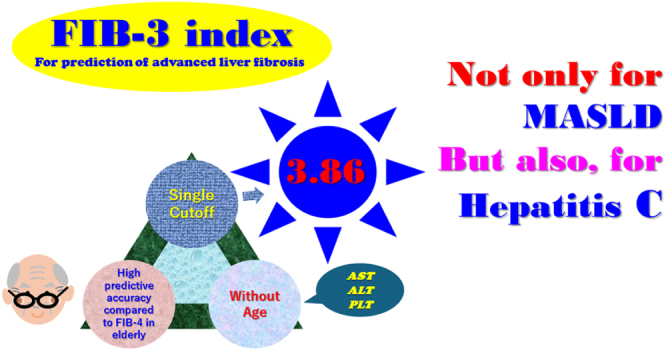

## Introduction

Chronic liver diseases have an increased rate of hepatic carcinogenesis as fibrosis progresses^[1,2]^. Evaluating the degree of liver fibrosis is crucial for predicting the prognosis. Although the incidence of carcinogenesis from viral liver diseases is decreasing due to direct-acting antiviral drugs for hepatitis C and nucleoside analogs for hepatitis B in Japan^[3]^, some cases of carcinogenesis originating from hepatitis B and C are still observed^[3]^.

Liver biopsy is the gold standard for evaluating the progression of liver fibrosis^[4]^. However, it carries the risk of complications and cannot be repeatedly performed^[4]^. Alternative methods such as Fibroscan® and MR elastography can be used^[5–7]^, but they are available only at some facilities and have limited applicability. Liver fibrosis can be easily evaluated using noninvasive liver fibrosis prediction scores (noninvasive test), such as the Fibrosis-4 index (FIB-4 index) and aspartate aminotransferase to platelet ratio index (APRI)^[8–12]^. However, the FIB-4 index includes age as a factor that leads to false-positive results in elderly patients^[13]^.

**HIGHLIGHTS**

- FIB-3 index demonstrates comparable performance to FIB-4 for predicting liver fibrosis in patients with hepatitis C.
- FIB-3 index enables fibrosis prediction using a single cutoff value regardless of age.
- FIB-3 index achieved superior diagnostic accuracy compared to FIB-4 in elderly patients (≥70 years).
- Both indices showed higher values in hepatitis C compared to MASLD cases due to lower platelet counts.
- Age-independent FIB-3 index offers enhanced clinical practicality by avoiding age-related false positives.

To address this limitation, the Fibrosis-3 (FIB-3) index^[14,15]^ was developed, which does not include age as a factor. Previously, we reported that the FIB-3 index has equal or superior utility to the FIB-4 index in metabolic dysfunction-associated steatotic liver disease (MASLD). However, the usefulness of the FIB-3 index for viral liver disease remains unclear.

This study aimed to investigate the effectiveness of FIB-3 index as a liver fibrosis prediction score in patients with hepatitis C.

## Methods

This retrospective observational cohort study has been reported in accordance with the STROBE (Strengthening the Reporting of Observational Studies in Epidemiology) guidelines for observational studies^[16]^.

### Patients

This retrospective observational study included consecutive treatment-naïve patients with chronic hepatitis C selected from an existing database of 517 patients who underwent liver biopsy at a municipal hospital between 1990 and 2023. Inclusion criteria were (1) histologically confirmed chronic hepatitis C, (2) treatment-naïve status, and (3) availability of complete laboratory data (aspartate transaminase, alanine transaminases, platelet count) required for FIB-3 and FIB-4 index calculations. Patients with incomplete laboratory parameters were excluded from the analysis. The database was anonymized and used in other studies.

The study protocol followed the ethical guidelines of the World Medical Association Declaration of Helsinki and was approved by the Institutional Review Boards of our hospital.

### Clinical assessment

Liver biochemistry values were determined in venous blood samples collected after an 8-h fast. The available parameters were used to compute the FIB-3 and FIB-4 indices^[9,14]^. The FIB-3 index was calculated as follows:

FIB-3 index = 5ln (AST [U/L]) − 2ln (ALT [U/L]) − 0.18(PLT [×10^4^/μL]) − 5^[14]^.

Liver biopsy samples were acquired under ultrasonographic guidance. Subsequently, formalin-fixed paraffin-embedded liver sections were stained with hematoxylin, eosin, or azan. The staging of liver fibrosis was performed by an expert pathologist.

The main evaluation endpoints were as follows:

1. FIB-3/FIB-4 index values at each fibrosis grade for patients with hepatitis C.
2. The liver fibrosis predictive ability of the FIB-3 index in patients with hepatitis C was investigated using receiver operating characteristic (ROC) analysis and scatter plots compared with the FIB-4 index.
3. Prediction of advanced liver fibrosis (≥F3) in hepatitis C virus (HCV) cases by the FIB-3/FIB-4 index by age-group using the single ideal cutoff value.

### Statistics

Continuous variables are presented as medians and ranges, except for the analysis of the FIB-3 and FIB-4 indices at each fibrosis stage, which were expressed as the mean ± standard deviation. Continuous and categorical variables were compared and examined using the Mann–Whitney *U* and Fisher’s exact tests, respectively.

The ideal cutoff values in the ROC analysis were determined using the Youden index. We performed a two-tailed *t*-test with the area under the ROC curve (AUROC) to assess statistical significance between the ROC curves. The Kaplan–Meier method and log-rank test were used for prognosis analysis.

Statistical significance was set at *P* < 0.05. All statistical analyses were performed using EZR (Saitama Medical Center, Jichi Medical University, Saitama, Japan)^[17]^ and R (The R Foundation for Statistical Computing, Vienna, Austria)^[18]^.

## Results

### Patient characteristics

Patient characteristics are shown in Table 1. The median patient age was 59 years, and 50.3% of the patients were male. Fibrosis stages 0, 1, 2, 3, and 4 were found in 3.9%, 58.4%, 26.7%, 9.4%, and 1.6%, respectively.

**Table 1:** Patient characteristics

Factor	HCV
*n*	517
Age	59 (20, 82)
Sex, male (%)	260 (50.3)
Observation period, years	12.3 (0.0, 18.9)
Activity stage (%)	
1	268 (53.1)
2	202 (40.0)
3	35 (6.9)
Fibrosis stage (%)	
0	20 (3.9)
1	298 (58.4)
2	136 (26.7)
3	48 (9.4)
4	8 (1.6)
Platelet (/μL)	18.4 (6.1, 64.6)
Albumin (g/dL)	4.30 (2.70, 67.20)
Aspartate aminotransferase (U/L)	43 (12, 2403)
Alanine aminotransferase (U/L)	46 (7, 4032)
Total bilirubin (mg/dL)	0.60 (0.20, 5.90)
Alpha-fetoprotein (ng/mL)	3.20 (0.80, 426.20)

Data are expressed as median (range) or number.

### FIB-3 and FIB-4 indices at each fibrosis stage

**Figure 1.**
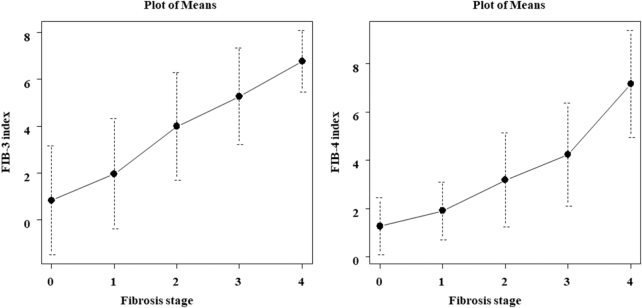
FIB-3 and FIB-4 indices at each fibrosis stage. The FIB-3 index increased linearly as fibrosis progressed, and the mean values ± standard deviations for each fibrosis stage were 0.8 ± 2.3, 1.96 ± 2.3, 3.9 ± 2.3, 5.2 ± 2.0, and 6.7 ± 1.3 for F0, F1, F2, F3, and F4, respectively. In contrast, the FIB-4 index increased exponentially as the fibrosis stage progressed, and the mean values ± standard deviations for each fibrosis stage were 1.2 ± 1.1, 1.9 ± 1.2, 3.1 ± 1.9, 4.2 ± 2.1, and 7.1 ± 2.2 for F0, F1, F2, F3, and F4, respectively. Error bars indicate standard deviation.

Figure 1 shows the distribution of FIB-3 and FIB-4 indices at each fibrosis stage. The means ± standard deviations of the FIB-3 index at F-0, -1, -2, -3, and -4 were 0.8 ± 2.3, 1.9 ± 2.3, 3.9 ± 2.3, 5.2 ± 2.1, and 6.7 ± 1.3, respectively. The corresponding values for the FIB-4 index were 1.2 ± 1.1, 1.9 ± 1.2, 3.1 ± 1.9, 4.2 ± 2.1, and 7.1 ± 2.2, respectively. Both indices increased linearly with the progression of fibrosis (Fig. 1).

### Abilities of the two indices in predicting advanced fibrosis (≥F3) and moderate fibrosis (≥F2)

ROC analysis was performed to compare the predictive abilities of FIB-3 and FIB-4 indices for advanced fibrosis (≥F3) and moderate fibrosis (≥F2) (Table 2).

**Table 2:** Receiver operating characteristic (ROC) curve analysis for liver fibrosis prediction using FIB-3 and FIB-4 indices

	≥F3			≥F2		
	Ideal cutoff	AUROC	*P*-value	Ideal cutoff	AUROC	*P*-value
FIB-3 index	3.86	0.79 (0.741–0.848)	0.975	2.24	0.790 (0.749–0.830)	0.423
FIB-4 index	2.50	0.79 (0.739–0.850)		2.10	0.781 (0.740–0.822)	

The cutoff values were calculated using the Youden index in the ROC analysis for each patient group. AUROC, area under the receiver operating characteristic curve; FIB-3 index, Fibrosis-3 index; FIB-4 index, Fibrosis-4 index.

The FIB-3 index demonstrated nearly equivalent fibrosis prediction capability to that of the FIB-4 index for predicting advanced (AUROC: 0.794 for FIB-3 vs 0.795 for FIB-4) and moderate liver fibrosis (AUROC: 0.790 for FIB-3 vs 0.781 for FIB-4) (Table 2).

**Figure 2.**
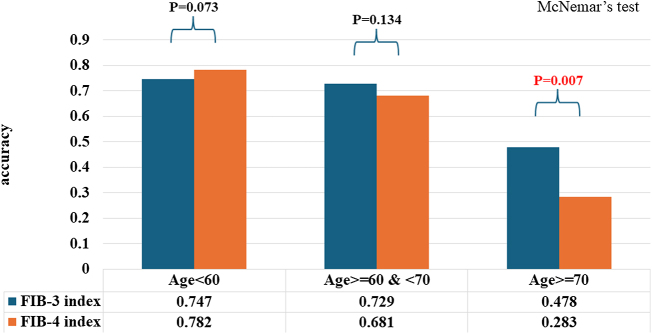
Diagnostic accuracy for advanced liver fibrosis by age-group by using a single cutoff value. We evaluated the diagnostic accuracy of the FIB-3 and FIB-4 indices for advanced hepatic fibrosis by age-group (<60, 60–69, and ≥70 years). The FIB-3 index demonstrated significantly superior diagnostic accuracy (0.478) compared to the FIB-4 index (0.283) in the group aged ≥70 years.

Furthermore, we evaluated the diagnostic accuracy of the FIB-3 and FIB-4 indices for advanced liver fibrosis (≥F3) by age-group (<60 years, 60–69 years, and ≥70 years) using a single cutoff value derived from the Youden index for the entire cohort. The FIB-3 index demonstrated significantly superior diagnostic accuracy (0.478) compared to the FIB-4 index (0.283) in the group aged ≥70 years (Fig. 2).

### Scatterplot of FIB-3 and FIB-4 indices by age

**Figure 3.**
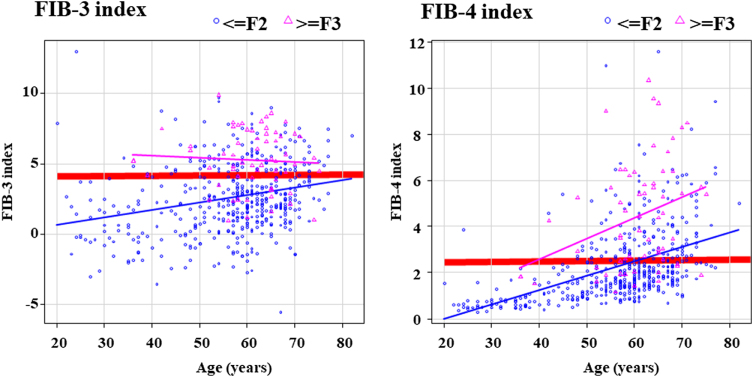
Scatter plot of FIB-3 and FIB-4 indices by age. Scatter plots demonstrate that both scores can distinguish between groups with and without ≥F3 fibrosis. The FIB-3 index can differentiate across all age-groups using a single cutoff value (3.86). Conversely, the FIB-4 index cannot distinguish the presence or absence of progressive fibrosis using only the ideal cutoff value of 2.50, necessitating age-specific cutoff values. Pink triangles (△) represent advanced liver fibrosis (≥F3), whereas blue circles (○) indicate absence of advanced liver fibrosis (≤F2). The pink and blue lines represent least-squares lines for each group. The red line indicates the ideal cutoff value derived from the Youden index.

To investigate the impact of age on fibrosis prediction using the FIB-3 and FIB-4 indices, we plotted age on the *x*-axis against the respective scores on the *y*-axis, stratifying the data according to the presence of ≥F3 fibrosis (Fig. 3).

Scatter plots demonstrated that both scores could distinguish between groups with and without ≥F3 fibrosis in patients with HCV. Although the FIB-3 index can differentiate across all age-groups using a single cutoff value, the FIB-4 index scores increased with age in both groups with and without advanced liver fibrosis. Differentiating between these two groups using the cutoff value (red line: 2.50) derived from the Youden index is challenging in individuals aged ≥60 years.

### Mean values of FIB-3 and FIB-4 indices for each liver fibrosis stage from each study

The mean values of the FIB-3 index for each hepatic fibrosis stage in MASLD from previous reports^[14,15]^ were extracted and compared with those of current HCV cases. Compared with the original^[14]^ and validation studies^[15]^ in MASLD cases, the FIB-3 index was 1–2 points higher for each stage (F0/1/2/3/4:0.8/2.0/4.0/5.3/6.8) in HCV cases (Table 3). Conversely, the FIB-4 index was 1–3 points higher (F0/1/2/3/4:1.3/1.9/3.2/4.2/7.2) in HCV cases than in both studies (Table 3).

**Table 3:** The mean values of FIB-3 and FIB-4 indices for advanced liver fibrosis from each study

	Fibrosis stage	GHA	HR	HCV
Median age of patients		55	57	59
Median platelet (/× 10^4^μL)		20.4	21.4	18.4
Median aspartate aminotransferase (U/L)		37	51	43
Median alanine aminotransferase (U/L)		58	73	46
FIB-3 index	F0	0.2	0.3	0.8
	F1	1.1	1.6	2.0
	F2	2.0	3.0	4.0
	F3	3.0	4.5	5.3
	F4	4.3	5.2	6.8
FIB-4 index	F0	1.1	1.1	1.3
	F1	1.3	1.5	1.9
	F2	1.9	2.3	3.2
	F3	2.4	3.5	4.2
	F4	4.1	5.2	7.2

In HCV cases, both FIB-3 and FIB-4 index scores for each fibrosis stage were 1–3 points higher than those in MASLD cases (GHA, Gastro-Hep Advances. 2022;1:1108–1113. Reference number 14: original study; HR, Hepatology Research. 25 April 2024. doi: 10.1111/hepr.14039; reference number 15: validation study). FIB, fibrosis.

## Discussion

Our key findings were as follows: the FIB-3 index, similar to the FIB-4 index, proved useful for predicting fibrosis in patients with untreated hepatitis C, whereas the FIB-4 index required age-specific cutoff values, the FIB-3 index allowed for fibrosis prediction using a single cutoff value across all age-groups.

In this study, we examined the utility of both the FIB-3 and FIB-4 index scores in patients with untreated hepatitis C, and both scores increased with disease progression. In HCV cases, both the FIB-3 and FIB-4 index scores for each fibrosis stage were 1–3 points higher than those in MASLD cases. These differences may be due to the disparity in platelet counts between HCV and MASLD cases at the same fibrosis stage. Generally, HCV cases tend to have lower platelet counts than MASLD cases at the same stage^[19]^. Consequently, both the FIB-3 and FIB-4 indices, which directly incorporate platelet count as a factor, yielded higher scores in HCV cases. As the FIB-4 index scores increase exponentially with liver fibrosis progression, the mean scores for the F4 stage show a greater difference between MASLD (4.1–5.2) and HCV cases (7.2) compared with the FIB-3 index (4.3–5.2 vs 6.8)^[14,15]^. Further data accumulation is required to confirm this observation.

The evaluation of advanced hepatic fibrosis prediction using ideal cutoff values derived from the Youden index across age-groups revealed that, although the diagnostic accuracy of the FIB-3 index slightly decreased with increasing age, the diagnostic accuracy of the FIB-4 index substantially declined as age increased, highlighting the need to adjust cutoff values based on age for the FIB-4 index to address this issue. The primary problem was the inclusion of age as a factor in the FIB-4 index.

The scatter plot (Fig. 3) further elucidates this issue. The FIB-3 index, which is normally distributed, was plotted directly, demonstrating that a single cutoff value can differentiate between the presence and absence of advanced hepatic fibrosis regardless of age. Conversely, the FIB-4 index, following an exponential distribution, was logarithmically transformed to an approximately normal distribution before plotting. However, a single cutoff value of the FIB-4 index cannot effectively separate the groups with and without advanced hepatic fibrosis because the scores for both groups increase with age, which represents a significant advantage of the FIB-3 index over the FIB-4 index.

Recent evidence has demonstrated the utility of the FIB-3 index for predicting liver fibrosis 5 years after achieving sustained virological response (SVR) in patients with chronic hepatitis C^[20]^. These findings in posttreatment HCV patients complement the current results in treatment-naïve patients, suggesting that the FIB-3 index maintains its predictive value across different stages of HCV management. This consistency across treatment phases supports the robustness of the FIB-3 index as a fibrosis assessment tool throughout the clinical course of HCV infection.

The age-independent nature of the FIB-3 index observed in both treatment-naïve and post-SVR populations may be particularly valuable in clinical practice, where patients of various ages and treatment stages require fibrosis assessment. The ability to use a single cutoff value regardless of age could simplify clinical decision-making and reduce the risk of age-related false positives across the entire spectrum of HCV care.

This study had several limitations. First, the database was from a single institution, and the diagnosis of fibrosis may vary among pathologists. Second, F4 cases were a few because liver biopsies are rarely performed in clinical practice. Further validation using noninvasive methods, such as FibroScan or MRE, is desirable. Additionally, this study focused on untreated HCV cases and lacked validation in HCV-SVR cases. The usefulness of the FIB-3 index in these populations requires further investigation, preferably using noninvasive methods such as FibroScan or MRE, because liver biopsies are rarely performed in these cases.

Future studies should focus on validating the FIB-3 index in other liver diseases and posttreatment HCV patients through multicenter trials. The development of disease-specific cutoff values and integration with other noninvasive methods warrant further investigation. Ultimately, the age-independent nature of the FIB-3 index suggests its potential for incorporation into clinical practice guidelines as a standardized fibrosis assessment tool.

In conclusion, we have demonstrated that the FIB-3 index is a useful tool for predicting liver fibrosis and prognosis in patients with hepatitis C. Moreover, its age-independent nature and ability to use a single cutoff value across all age-groups make it a potentially valuable tool in clinical practice.

## Data Availability

The data are not publicly available due to privacy restrictions.

## Research registration unique identifying number (UIN)

researchregistry11294.
